# Prediction of postoperative cardiac events in multiple surgical cohorts using a multimodal and integrative decision support system

**DOI:** 10.1038/s41598-022-15496-w

**Published:** 2022-07-05

**Authors:** Renaid B. Kim, Olivia P. Alge, Gang Liu, Ben E. Biesterveld, Glenn Wakam, Aaron M. Williams, Michael R. Mathis, Kayvan Najarian, Jonathan Gryak

**Affiliations:** 1 Department of Computational Medicine and Bioinformatics, University of Michigan, Ann Arbor, MI 48109 USA; 2 Department of Surgery, University of Michigan, Ann Arbor, MI 48109 USA; 3 Department of Anesthesiology, University of Michigan, Ann Arbor, MI 48109 USA; 4 Michigan Institute for Data Science (MIDAS), University of Michigan, Ann Arbor, MI 48109 USA; 5 Michigan Center for Integrative Research in Critical Care (MCIRCC), University of Michigan, Ann Arbor, MI 48109 USA

**Keywords:** Data integration, Machine learning, Predictive medicine

## Abstract

Postoperative patients are at risk of life-threatening complications such as hemodynamic decompensation or arrhythmia. Automated detection of patients with such risks via a real-time clinical decision support system may provide opportunities for early and timely interventions that can significantly improve patient outcomes. We utilize multimodal features derived from digital signal processing techniques and tensor formation, as well as the electronic health record (EHR), to create machine learning models that predict the occurrence of several life-threatening complications up to 4 hours prior to the event. In order to ensure that our models are generalizable across different surgical cohorts, we trained the models on a cardiac surgery cohort and tested them on vascular and non-cardiac acute surgery cohorts. The best performing models achieved an area under the receiver operating characteristic curve (AUROC) of 0.94 on training and 0.94 and 0.82, respectively, on testing for the 0.5-hour interval. The AUROCs only slightly dropped to 0.93, 0.92, and 0.77, respectively, for the 4-hour interval. This study serves as a proof-of-concept that EHR data and physiologic waveform data can be combined to enable the early detection of postoperative deterioration events.

## Introduction

Patients who undergo cardiovascular surgery are at risk of developing hemodynamic decompensation^1^. Decompensation can include arrhythmias (e.g., atrial fibrillation), hypotension, and pulmonary edema. Monitoring devices in intensive care units (ICU) produce wealth of data, but the deluge of information produced, in addition to false alarms, alarm fatigue, and cognitive biases, can negatively affect patient care^2–4^. As such, there is a growing need for predictive models and effective clinical decision support systems (CDSS) that identify only the relevant data from multiple sources to give information to healthcare providers.

Previous work has incorporated electronic health record (EHR) data in machine learning (ML) to create predictive models, as EHR data are noninvasive and routinely collected. Some examples of previous studies include^5^ to predict coronary artery disease^6^, to compute survival risk scores, and^7–9^ to predict mortality or 30-day readmission.

Other research focuses on the utility of physiological signals. Several models^10–12^ used features from heart rate variability (HRV) to predict mortality or vascular events. Belle et al.^13^ showed that HRV and electrocardiogram (ECG) features could better predict hemodynamic stability compared to standard biomarkers used in hemodynamic assessment. Hernandez et al.^14^ took a multimodal approach, using ECG, HRV, arterial blood pressure (ABP) waveform from an arterial line, pulse plethysmography (PPG) waveform from a pulse oximeter, and EHR data to predict hemodynamic decompensation.

In this study, we use multimodal features from physiological signals and EHR data to create ML models that predict hemodynamic decompensation after surgery in surgical ICU patients. We do this in order to test the hypothesis that models trained on a cardiovascular surgery cohort may be successfully applied to other cohorts that underwent different surgical procedures (vascular and acute non-cardiac surgery). We then share the results of applying the models trained on the single surgical cohort to the other postoperative cohorts, which show that the models are robust and can be generalized to the other postoperative patient groups.

## Methods

**Figure 1:**
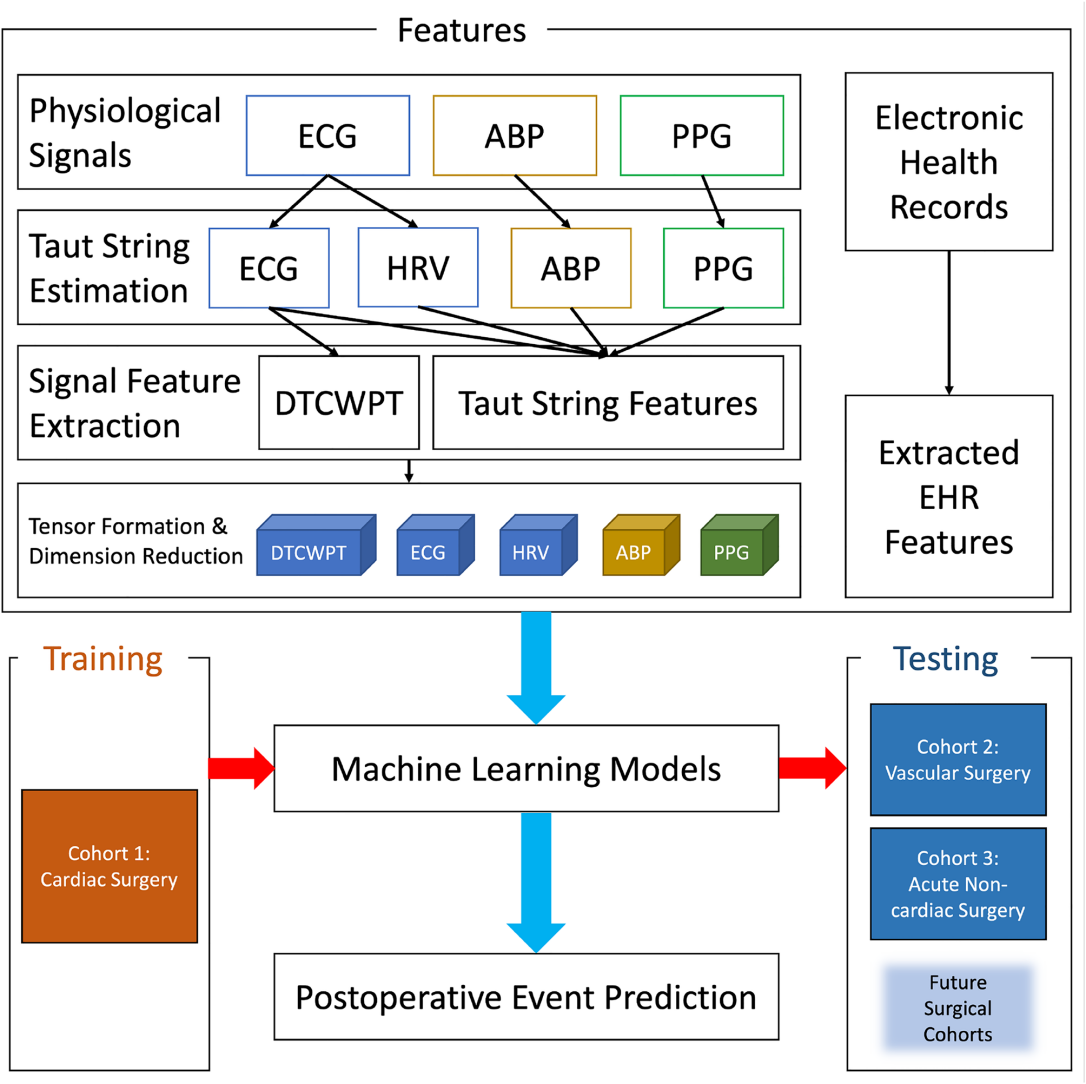
A schematic of the utilized methods. ECG: Electrocardiogram; ABP: Arterial Blood Pressure; PPG: Pulse Plethysmography; HRV: Heart Rate Variability; DTCWPT: Dual-tree Complex Wavelet Packet Transform; TS: Taut String.

This section details the creation of the predictive models for hemodynamic decompensation, which builds upon our previous work^14–16^. A graphical schematic of the method is presented in Fig. 1.

### Dataset

Michigan Medicine data systems provided the EHR data and physiological signal data, including ECG, ABP, and PPG waveform data. Our data include features extracted from Taut String estimates of ECG, ABP, PPG, and HRV, dual-tree complex wavelet packet transform (DTCWPT) features extracted from the Taut String estimation of ECG waveform, and features extracted from EHR data from three postoperative cohorts of surgical patients. The Taut String and DTCWPT methods of feature extraction are described in further detail in the "Signal processing for feature extraction" section, and the list of EHR data features is included in the Supplementary Information.

#### Patient cohorts

The three cohorts of surgical patients have increasing heterogeneity. Cohort 1 consists of patients recovering from elective cardiac surgery, Cohort 2 major vascular surgeries, and Cohort 3 acute conditions requiring urgent and/or major non-cardiac surgery. Examples of surgeries in Cohort 1 include coronary artery bypass grafting, cardiac valve repair/replacement, and thoracic aortic procedures; Cohort 2 abdominal aortic aneurysm surgeries (both open repairs and endovascular stenting) and major vascular bypass procedures (e.g., aortofemoral bypass, axillofemoral bypass, and femoral-popliteal artery bypass); Cohort 3 major abdominal surgeries (e.g., exploratory laparotomies), orthopedic surgeries (e.g., total hip replacements for hip fractures), and neurosurgeries (e.g., craniotomies and spinal fusions).

Seven adverse events associated with hemodynamic decompensation are included in our study: low cardiac index, sustained low mean arterial pressure, epinephrine bolus, inotropic therapy initiated, inotropic therapy escalated by $\ge$100%, vasopressor therapy initiated, and vasopressor therapy escalated by $\ge$100%. In addition, we include two additional adverse events that may not necessarily results from hemodynamic decompensation but are clinically significant enough to warrant detection by an algorithm: re-intubation and mortality, as an algorithm missing these two events while detecting other decompensation events would be clinically less meaningful. The exact definitions, details, and rationale for inclusion of these events as agreed by our clinical team are available in Hernandez et al.^14^.

### Signal processing for feature extraction

We divided ECG, ABP, and PPG signals into five non-overlapping tumbling windows three minutes in length. We created four prediction windows of different lengths: 30 minutes, 1 hour, 2 hours, and 4 hours.

#### Data preprocessing

To remove artifacts, each ECG tumbling window was preprocessed using a second order Butterworth bandpass filter with cutoff frequencies of 0.5 and 40 Hz. Similarly, each ABP window was preprocessed with a third order Butterworth bandpass filter with cutoff frequencies 1.25 and 25 Hz, and each PPG window with a third order Butterworth bandpass filter with cutoff frequencies 1.75 and 10 Hz.

#### Heart rate variability

We identified peaks within the filtered ECG signal using the peak detection method defined in Hernandez et al.^14^. We compute the difference in time between subsequent peaks to produce HRV.

#### Taut string

Taut String (TS)^17^ has previously been used to capture hemodynamic instability in ECG^13,14^, and we utilize these same TS features in our method with varying values for $\epsilon$.

Given a discrete signal $f = (f_1, f_2, ..., f_n)$, we define the first-order finite difference as

1 $$\begin{aligned} \text {diff}(f) = (f_2 - f_1, ..., f_n - f_{n-1}). \end{aligned}$$

We use a parameter $\epsilon > 0$ to create the piecewise linear function *g*, the TS estimate of *f*, such that the max norm of $(f-g)$ is $\le \epsilon$ and the Euclidean norm of $\text {diff}(g)$ is minimal, defined below as

2 $$\begin{aligned} \Vert f-g\Vert _\infty = \max _i \{ |f_i - g_i |\} \le \epsilon \end{aligned}$$

and

3 $$\begin{aligned} \Vert \text {diff}(g)\Vert _2 = \sqrt{\sum _{i=1}^{n-1} (x_{i+1} - x_i)^2}, \end{aligned}$$

respectively.

Next, $\text {diff}^*$ is defined as

4 $$\begin{aligned} \text {diff}^* (y_1, y_2, ..., y_{n-1}) = (-y_1, y_1 - y_2, ..., y_{n-2} - y_{n-1}, y_{n-1}). \end{aligned}$$

Here, $\text {diff}^* : \mathbb {R}^{n-1} \rightarrow \mathbb {R}^n$ is dual to $\text {diff} : \mathbb {R}^n \rightarrow \mathbb {R}^{n-1}$. The function *g* also minimizes

5 $$\begin{aligned} \Vert \text {diff}^* \text {diff}\Vert _1 = |g_2 - g_1 |+ \sum ^{n-1}_{i=2} |g_{i-1} - 2g_i + g_{i+1} |+ |g_n - g_{n-1} |. \end{aligned}$$

**Figure 2:**
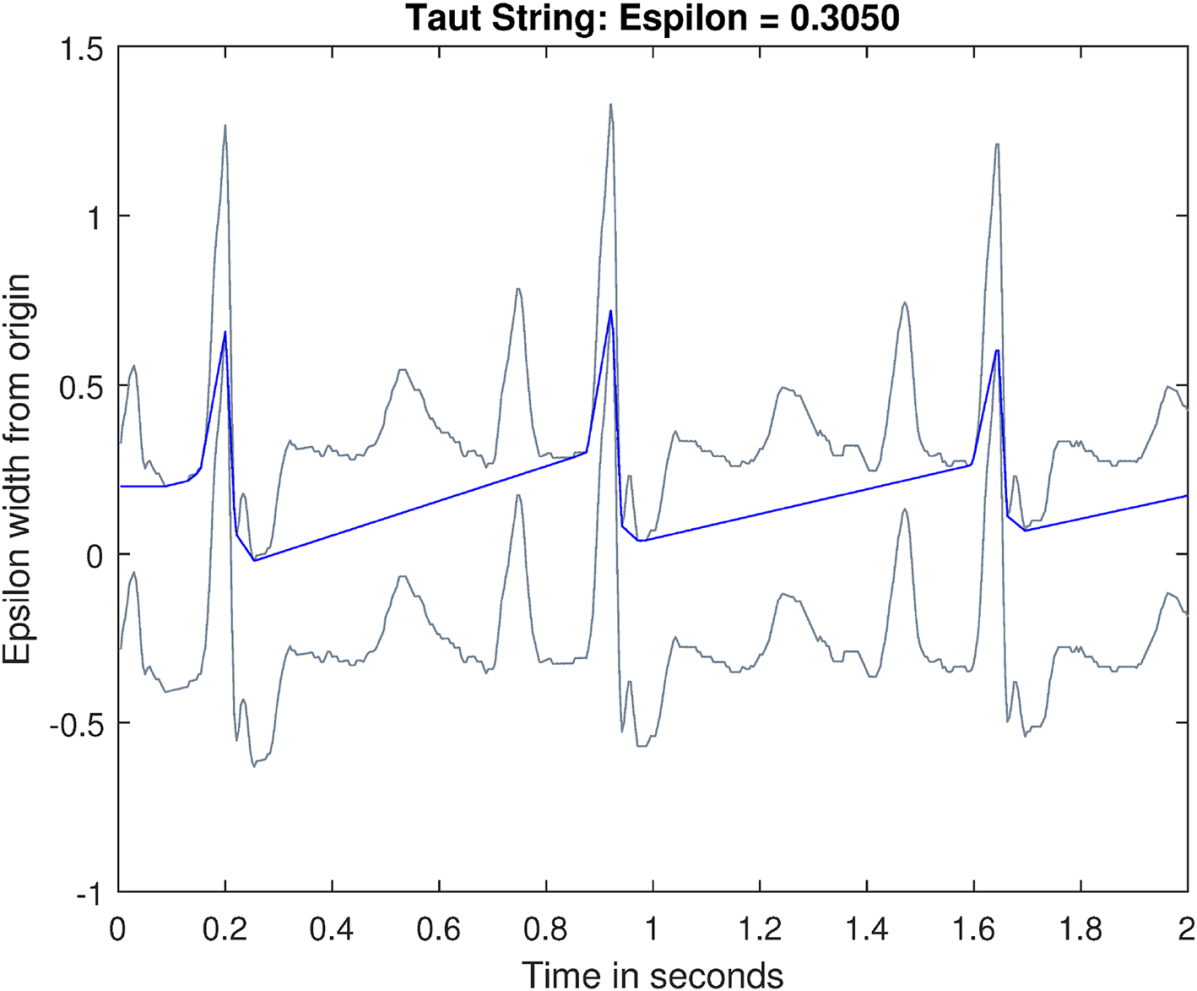
TS Approximation of ECG Signal. The grey waveforms are the margins for TS, $f + \epsilon$ and $f - \epsilon$, the blue line is the TS estimation.

One can visualize the function *g* as a string pulled tightly between $f + \epsilon$ and $f - \epsilon$. It is generally a piecewise linear function that results in a smoother line than *f*. Figure 2 shows a sample TS estimate of ECG.

We applied TS to each tumbling window of ECG, HRV, ABP, and PPG signals with different $\epsilon$ values. A summary of TS estimations performed and the number of features extracted are listed in Table 1. These features are detailed in Hernandez et al.^14^.

**Table 1:** Taut string estimation information.

(a) Epsilon values for each feature type	
Feature type	$\varepsilon$ values
ECG	0.0100, 0.1575, 0.3050, 0.4525, 0.6000
HRV	0.0010, 0.0258, 0.0505, 0.0753, 0.1000
DTCWPT	0.0100, 0.1575, 0.3050, 0.4525, 0.6000
ABP	0.1000, 0.7000, 1.3000, 1.9000, 2.5000
PPG	1.0000, 8.7500, 16.5000, 24.2500, 32.0000

(b) Features extracted per epsilon value	
Feature type	Number of features extracted
ECG	6
HRV	6
DTCWPT	152
ABP	21
PPG	21

#### Dual-tree complex wavelet packet transform

Dual-tree complex wavelet packet transform (DTCWPT)^18^ has previously been used in an ECG context^14^. More detail is available in Bayram et al.^18^, but briefly: at each level *k*, DTCWPT uses a high- and low-pass perfect reconstruction wavelet filter bank to decompose the previous level’s subbands. Increasing *k* yields increased frequency resolution, but at computational expense. We select $k=2$ in this study.

The filter banks of *k* are selected such that the first filter bank’s discrete Hilbert transform is the frequency response of each branch of the second filter bank. This allows for approximate shift-invariance. If $\Psi$ is the wavelet for low-pass filter $h_0(n)$ and high-pass filter $h_1(n)$, and $\Psi ^{\prime }$ or $\mathscr {H} \{ \Psi \}$ is its Hilbert pair, then the *z*-transforms of the two filters, $H_0$ and $H_1$, are related by

6 $$\begin{aligned} H_1(e^{jw}) = -e^{jdw}H_0^{*}(e^{j(w-d)}) \end{aligned}$$

when the wavelet basis is orthonormal. $H_1$ and $H_1^{\prime }$ have the relationship

7 $$\begin{aligned} H_1^{\prime } = -j \cdot \text {signum}(w)e^{j0.5w}H_1(e^{jw}), \end{aligned}$$

where *d* is an odd integer and $|w |< \pi$.

Following this, if $H^{(k)}(e^{jw})$ is the equivalent response at level *k*, then:

8 $$\begin{aligned} H^{(k)}(e^{jw}) = H_1(e^{j2(k-1)w}) \prod ^{k-2}_{m=0}H_0(e^{j2mw}) \end{aligned}$$

and the equivalent response of the second filter bank’s corresponding branch is

9 $$ H^{\prime } (k)(e^{{jw}} ) =  - e^{{j0.5w}} {\mathcal{H}}\left\{ {H^{{(k)}} (e^{{jw}} )} \right\} $$

according to^18^.

### Electronic health records

EHR data was available for each subject in addition to physiological signals. It included static information, including age, race, and comorbidities, as well as temporal information, such as lab results and medications administered. The features that required different levels of representation through one-hot encoding are presented in Supplementary Tables S1 through S4 and also further explained in Hernandez et al.^14^. For completeness, the temporal EHR information for each tumbling window was carried over from the most recent record.

### Tensor formation and reduction

Taking into account all values of $\epsilon$ from TS estimation, the filter banks of DTCWPT, and the five tumbling windows, a total of 5,150 features are extracted from ECG, HRV, ABP, and PPG signals. To reduce the feature space of this data and maintain structural information, we rely on tensor decomposition.

First, we format the data into a tensor. Each type of signal features has the structure of five tumbling windows and five values of $\epsilon$, with varying numbers of features. We standardize the features within each tumbling window and $\epsilon$ value using mean and standard deviation (SD) of each $\epsilon$-window-feature entry from the training set. These training set values are later used to standardize the $\epsilon$-window-feature entries from the test set. We construct a third-order tensor of structure ($\epsilon \times \text {feature} \times \text {window}$) for each feature type, visualized in Table 2. Once the tensors of a feature type have been created for all subjects in the training set, they are stacked along a new fourth mode, generating a tensor of size $(\epsilon \times \text {feature} \times \text {window} \times N_{\text {train}})$, where $N_{\text {train}}$ is the number of individuals in the training set. This is repeated for tensors of each feature type. We perform higher-order singular value decomposition (HOSVD)^19–21^ using *Tensor Toolbox*^22^ to reduce the DTCWPT, ABP, and PPG tensors to their core tensors, $G_{\text {DTCWPT}}, G_{\text {ABP}}, G_{\text {PPG}}$, and reserve their respective transformation matrices ($U_{\text {DTCWPT}}, U_{\text {ABP}}, U_{\text {PPG}}$).

**Table 2:** Tensors formed for each feature type.

Feature type	Tensor dimensions
ECG	$5 \times 6 \times 5$
HRV	$5 \times 6 \times 5$
DTCWPT	$5 \times 152 \times 5$
ABP	$5 \times 21 \times 5$
PPG	$5 \times 21 \times 5$

Next, we stack all fourth order tensors from the different feature types along the feature mode (second mode) to create a new tensor *T*. We then perform a Canonical Polyadic (CP) decomposition^23^ to obtain *T*’s four factor matrices: (*A*, *B*, *C*, *D*). Given the tensor $T = \mathbb {R}^{n_1 \times n_2 \times n_3 \times n_4}$ and rank *r*, the CP decomposition produces the tensor

10 $$\begin{aligned} \hat{T} = \sum ^{r}_{i=1} a_i \otimes b_i \otimes c_i \otimes d_i \end{aligned}$$

such that $\Vert T - \hat{T}\Vert$ is minimized. The tensor Frobenius norm $\Vert \cdot \Vert$ is defined as

11 $$\begin{aligned} \Vert X\Vert = \sqrt{ \sum _{i_1, ..., i_m} (x_{i_1 ... i_m})^2 }, \end{aligned}$$

where $x_{i_1 ... i_m}$ is the $(i_1, ..., i_m)$ entry of *X*.

We use alternating least squares (ALS)^23^ to solve the CP decomposition, setting rank $r=4$ as done in Hernandez et al.^14^. After solving for $\hat{T}$, we reserve factor matrices *A* and *C* for feature extraction.

Now that we have the transformation matrices from HOSVD and the factor matrices from CP-ALS, we can compute the core tensor of any DTCWPT, ABP, or PPG tensor in the test set. We perform the following to compute features for each individual *j* in the test set: Using the transformation matrices from HOSVD ($U_{\text {DTCWPT}}, U_{\text {ABP}}, U_{\text {PPG}}$), core tensors are computed for DTCWPT, ABP, and PPG. Next, all of *j*’s tensors are stacked along mode 2 to build the new tensor $S_j$. Lastly, the factor matrices *A* and *C* from *T*’s CP decomposition are used to solve for *B* via least squares by minimizing

12 $$\begin{aligned} \Vert S_j - B(C\odot A)^{\intercal }\Vert . \end{aligned}$$

The optimal solution is calculated as

13 $$\begin{aligned} B = S_{(2)} (C \odot A)(A^{\intercal }A * C^{\intercal }C)^{\dag }, \end{aligned}$$

where $S_{(2)}$ is the mode-2 slice of tensor $S_j$, $\odot$ is the Khatri-Rao product, and $\dag$ is the Moore-Penrose pseudoinverse^23^.

### Machine learning

The ML models are created to predict an occurrence of the nine adverse events detailed in the Data section. in prediction windows 0.5, 1, 2, and 4 hours before the event. The training set consisted of Cohort 1 while the test set was Cohort 2 or 3 exclusively. The incidence rates of adverse events by cohort and prediction window are available in Table 3. Given that clinicians may want to detect more cases with adverse outcomes at the risk of somewhat increased false positives, we balanced the training set to have a ratio of 0.35 to 0.65 between positive and negative cases by discarding excess negative cases. No such adjustment was performed for the test sets. Patients without complete signals in the analysis windows (e.g., ECG signals with missing R peaks) were excluded. The respective sample size for each prediction window is available in Table 4.

**Table 3:** Incidence rate of adverse outcomes in each cohort.

Prediction window (hrs)	Cohort 1	Cohort 2	Cohort 3
0.5	0.174	0.212	0.905
1	0.187	0.283	0.900
2	0.166	0.234	0.813
4	0.158	0.190	0.750

**Table 4:** Mean AUROC and standard deviation of the models.

Prediction window (hrs)	LUCCK	RF	NB	SVM
	Mean (SD)	Mean (SD)	Mean (SD)	Mean (SD)
**(a) Cohort 1 - Cardiac Surgery Cohort (Training Set)**				
0.5 ($n=423$)	0.90 (0.01)	**0.94** (0.01)	0.83 (0.02)	0.91 (0.01)
1 ($n=466$)	0.89 (0.01)	**0.93** (0.01)	0.84 (0.02)	0.91 (0.01)
2 ($n=426$)	0.89 (0.01)	**0.93** (0.01)	0.83 (0.02)	0.90 (0.01)
4 ($n=414$)	0.91 (0.01)	**0.93** (0.01)	0.85 (0.02)	0.91 (0.01)
**(b) Cohort 2 - Vascular Surgery Cohort (Test Set A)**				
0.5 ($n=66$)	**0.94** (0.02)	**0.94** (0.01)	0.82 (0.06)	0.91 (0.08)
1 ($n=60$)	**0.94** (0.02)	**0.94** (0.01)	0.83 (0.07)	0.91 (0.06)
2 ($n=64$)	0.92 (0.01)	**0.94** (0.02)	0.88 (0.04)	0.92 (0.04)
4 ($n=63$)	0.90 (0.02)	**0.92** (0.02)	0.87 (0.03)	0.90 (0.06)
**(c) Cohort 3 - Acute Non-cardiac Surgery Cohort (Test Set B)**				
0.5 ($n=21$)	0.75 (0.13)	**0.82** (0.15)	0.42 (0.03)	0.80 (0.15)
1 ($n=20$)	0.75 (0.12)	**0.82** (0.10)	0.51 (0.09)	0.67 (0.16)
2 ($n=16$)	0.74 (0.08)	0.72 (0.16)	0.79 (0.07)	**0.81** (0.16)
4 ($n=12$)	**0.77** (0.09)	**0.77** (0.15)	0.63 (0.10)	0.74 (0.14)

The means and standard deviations (SDs) of AUROC for models trained on the Cohort 1 (Cardiac Surgery) and tested on Cohorts 2 (Vascular Surgery) and 3 (Acute Non-cardiac Surgery). The *n* in each row represents the number of deterioration events (i.e., the sample size). The best performance in each prediction window is boldfaced.

Model training consisted of 3-fold cross-validation (CV) of Cohort 1 data to select optimal hyperparameters for each model using a validation set. Models were then trained on all three folds with the selected hyperparameters in Cohort 1, and tested against Cohort 2 and Cohort 3 data. This process was repeated 101 times, with shuffling of data across CV folds, for each model to obtain the mean area under the receiver operating characteristic curve (AUROC).

Naive Bayes (NB) models with normal distribution were trained with no hyperparameter tuning, serving as the simplest baseline models.

Random forest (RF) models^24^ were trained with varying numbers of trees (50, 75, or 100), minimum leaf size (1, 5, 10, 15, or 20), percentage of features to include for maximum number of splits (25, 50, 75, 100%), split criterion (Gini impurity or cross entropy), and number of predictors to sample ($[10,100]$ in increments of 10) using grid search to select hyperparameters.

Support vector machines (SVM)^25^ were trained with a linear kernel via sequential minimal optimization. Grid search was used to determine the optimal box constraint *C* and scaling parameter $\gamma$, where $C \in [10^{-7}, 10^{12}]$ and $\gamma \in [10^{-12}, 10^{12}]$ consist of logarithmically-spaced values.

Learning using concave and convex kernels (LUCCK) is a novel machine learning method developed in 2019^26^. This method is designed to maintain the robustness and generalizability of performance with a smaller dataset. For a feature space of vectors $\text {x} = (x_1, ..., x_n) \in \mathbb {R}^n$, there is a similarity function

14 $$\begin{aligned} Q(\text {x}) = \prod _{i=1}^{n}(1+\lambda _{i}x_{i}^{2})^{-\theta _i} \end{aligned}$$

where $\lambda _i > 0$ and $\theta _i > 0$. The range of $\lambda$ values used in hyperparameter optimization was [0.01, 0.1] in increments of 0.01, and the range of $\theta$ values was in [0.1,1.0], in increments of 0.1. We selected for $\lambda$ and $\theta$ via grid search.

Additionally, the mean Shapley values^27^ of all features across all training data from the best performing model were obtained to provide the model explainability. The Shapely value of a given feature indicates the deviation of prediction from the average prediction caused by the feature.

### Ethics declarations

The human data collection and use were approved by the University of Michigan’s Institutional Review Board (IRB) under HUM00092309. The informed consent was obtained and all experiments in this study were performed in accordance with this approval.

## Results

The AUROCs of each ML model are presented in Table 4.

### Cohort 1 - training with cardiac surgery cohort

During training with Cohort 1, the RF models exhibit the highest AUROCs between 0.93 and 0.94 across all gaps, followed by the SVM (0.90–0.91) and LUCCK (0.89–0.91). The NB models achieve the lowest performance (0.83-0.85).

### Cohort 2 - testing with vascular surgery cohort

The RF models achieve the highest AUROCs on testing with Cohort 2. The LUCCK models perform marginally *better* on Cohort 2 compared to the training set, achieving AUROCs similar to those of RF. The NB and SVM models also achieve AUROCs comparable with those obtained on the training set, with the NB models on 2- and 4-hour windows actually achieving *higher* AUROCs than their respective training sets. The NB and SVM models demonstrate higher SDs compared to the LUCCK and RF models.

### Cohort 3 - Testing with acute non-cardiac surgery cohort

With Cohort 3, the overall performance is lower across all models. The RF models achieve the highest performance, with the models on 0.5- and 1-hour windows achieving AUROCs greater than 0.8. LUCCK models maintain mean AUROCs between 0.74 and 0.77 across models on all windows, while the SVM’s AUROCs fluctuate to a larger extent, between 0.67 and 0.81. The NB models decline to the greatest extent, exhibiting an AUROC as low as 0.42 on the 0.5-hour window.

### Shapley values for the best performing model

**Figure 3:**
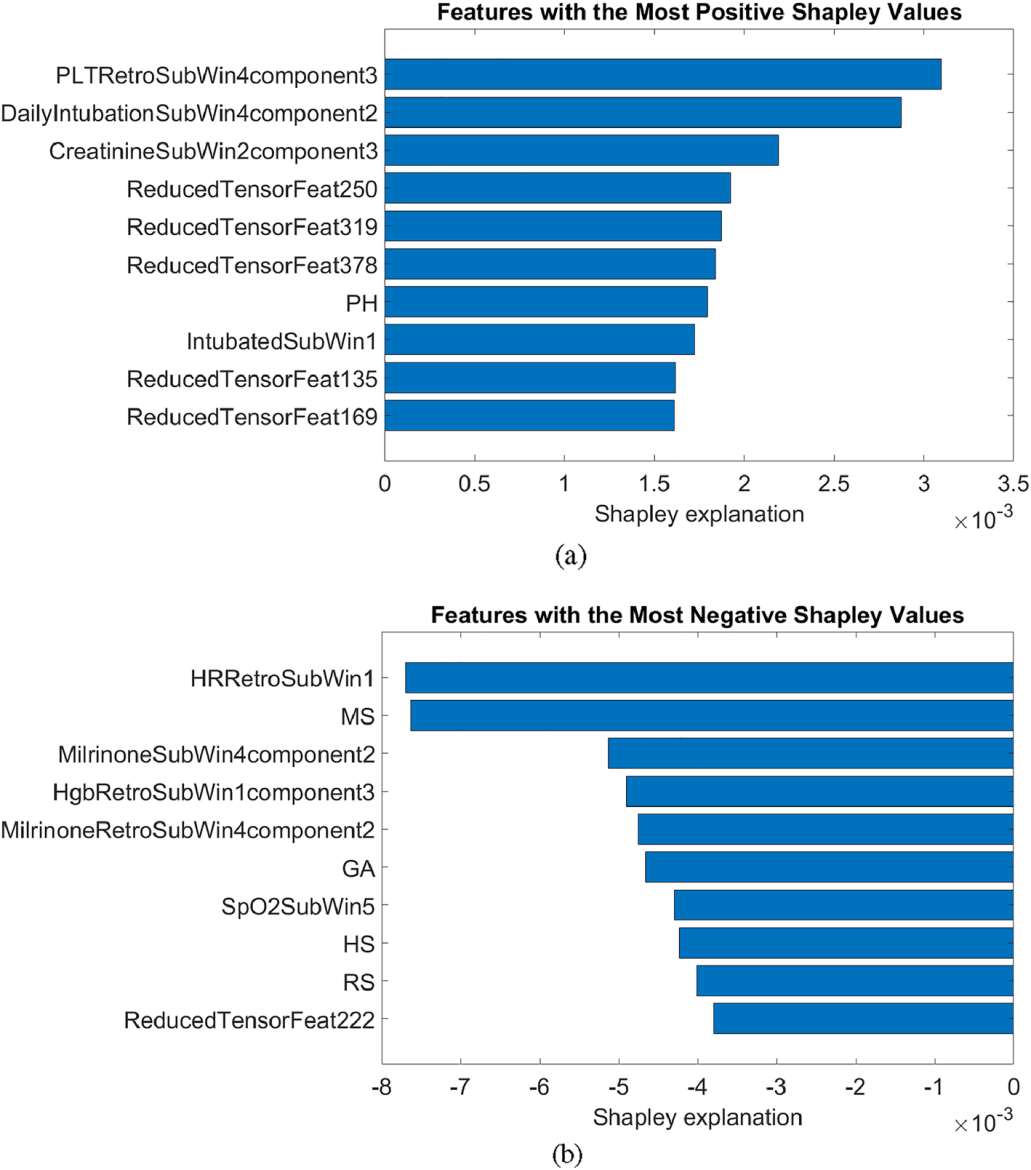
Features with the most positive and most negative Shapley values, 10 each. Features whose names start with “ReducedTensor” are reduced tensor signal features. “Retro” denotes retrospective features (Supplementary Tables 1–4). “SubWin” followed by a number indicates the *n*th tumbling or retrospective window. The information on what each component means for each feature is available in Supplementary Tables 1–4. PLT: Platelet counts. DailyIntubation: Whether or not the patient has been re-intubated. Intubated: the patient’s intubation status. HR: Heart Rate. Milrinone: Milrinone infusion. Hgb: Hemoglobin. SpO2: Oxygen saturation. PH, MS, GA, HS and RS are the number of medications in the given category administered during the patient’s hospital stay. PH: Pharmaceutical aids/reagents. MS: Musculoskeletal medications. GA: Gastrointestinal medications. HS: Hormones/synthetics/modifiers. RS: Rectal (local) medications.

The best performing model was the 36th Random Forest model with the 0.5-hour gap (AUROC: 0.9698). In this model, 384 tensor-reduced signal features (41%) and 542 EHR features (59%) were used. The 10 features each with the most positive and most negative Shapley values are presented in Fig. 3. In this model, 5 out of 10 features with the most positive Shapley values and 1 out of 10 features with the most negative Shapley values are tensor signal features.

## Discussion

In this study of postoperative deterioration events among three successively more heterogeneous cohorts of surgical patients, the incidence rates of a patient-level deterioration event were 16–19%, 19–28%, and 75–90%, respectively, although for Cohort 1, we adjusted the rate of positive cases to 35% during training. The best performing models for each cohort yielded AUROCs of 0.94, 0.94 and 0.82, respectively, all for 0.5-hour prediction windows. Our study serves as a proof-of-concept that EHR data and physiologic waveform data may be combined to improve the early detection of postoperative deterioration events, even when trained on one surgical cohort and applied to other cohorts.

The predictive performance is well maintained throughout all prediction windows during training with Cohort 1; the RF models achieve the highest performance. With Cohort 2, likely due to smaller sample size, the SDs of AUROCs of the models tend to be higher than those of the training set. Nonetheless, these models achieve performance comparable to that achieved on the training set, with some models (especially LUCCK) even exceeding the mean AUROCs achieved in training. Overall, the models trained on Cohort 1 generalize extremely well to the Cohort 2.

When the models are tested on Cohort 3, the acute non-cardiac surgery cohort, the performance of all models decline. This is likely due to the paucity of samples in Cohort 3, which is a fraction of Cohort 2, let alone Cohort 1; the SDs of the AUROCs are also much larger than their counterparts in Cohorts 1 and 2. The NB models suffer most from the performance decline, with models on 0.5- and 1-hour prediction windows performing near or worse than random guessing. Given the simplicity of the NB models, it is not surprising that they fail to generalize to a small dataset from a different surgical cohort.

On the other hand, the other three models maintain an acceptable performance for most prediction windows despite the paucity of data. The RF models achieve the highest AUROC with the 0.5- and 1-hour prediction windows at 0.82. The AUROC declines for the 2-hour prediction window to 0.72 but recovers to 0.77 at the 4-hour prediction window. The SVM model displays even more fluctuation, with the highest AUROC of 0.81 on the 2-hour prediction window and lowest AUROC of 0.67 on the 1-hour prediction window. The LUCCK models maintain the most consistent range of performance, between 0.74 and 0.77 across all prediction windows, with generally smaller SDs than RF and SVM. LUCCK also does not experience a steep decline that the RF and SVM models show in the 1- and 2-hour prediction windows, respectively. It should also be noted that LUCCK’s generalization with Cohort 2 was the best among the four models, showing the largest *increase* in performance from training when tested on Cohort 2. Such findings are consistent with previous reports indicating that the LUCCK models are capable of demonstrating more robust and generalizable performance, especially when the number of samples is small^14,26^, which is often the case with clinical data.

The Shapley values obtained from the best performing model indicate that both the signal and EHR features make meaningful contributions towards the prediction, as both categories of features are fairly represented in the lists of Shapley values with the highest magnitudes (Figure 3). A positive Shapley value indicates the importance of the feature with respect to the positive class (i.e., an occurrence of a deterioration event), while a negative value indicates the same for the negative class (i.e., the non-occurrence of a deterioration event). Also, we may also infer that the signal features are more represented in the marginal contribution towards the prediction of an occurrence rather than a non-occurrence of an adverse cardiovascular event, as indicated by the greater number of signal features with very positive Shapley values than very negative ones.

A few predictive scoring systems for assessing mortality risks in the ICU patients have been developed in clinical settings. The APACHE II scoring system, which utilizes 12 physiological measurements, age, and previous health conditions from 5030 ICU patients in 13 hospitals to create a logistic regression model to predict hospital death, reported an AUROC of 0.863^28^.

The SAPS II scoring system, which utilizes 12 physiological variables, age, type of admission, and three underlying disease variables to create a logistic regression model predicting the hospital mortality based on 12,997 ICU patients from 12 countries, reported an AUROC of 0.88 on the developmental dataset and 0.86 on the validation dataset^29^.

The evaluation of Sequential Organ Failure Assessment (SOFA) scoring system^30^, which utilizes physiological variables from various organ systems to assign a score between 0 and 24, demonstrated the highest AUROC of 0.90 based on 352 patients when using the highest SOFA score taken during the entire ICU stay.

Our study differs from these studies in two major aspects: (1) We have included features derived from several digital signal processing (DSP) techniques performed on the physiological waveform data, and (2) our models are trained to predict other clinical deterioration events besides mortality, given that the ultimate form of these models is intended for real-time clinical monitoring in a CDSS. Despite being trained on just a few hundred cases, our best-performing models can achieve AUROCs between 0.90 and 0.94 for all prediction windows on Cohort 2. Although Cohort 3’s best performing models achieve a maximum AUROC of 0.82, we expect that such performance can be improved upon including additional quality physiological waveform data, given the largest sample size in this Cohort was 21.

Monitoring patients in the ICU for cardiovascular adverse events and complications is a crucial component of postoperative critical care. Early warning systems for patients predicted to be at risk of major cardiovascular complications in advance of clinicians’ attention can enable potentially life-saving interventions to be pursued before deterioration onset and significantly improve clinical outcomes. However, because different surgical procedures imply different underlying patient pathologies, along with varying levels of invasiveness and resultant derangements to organ systems, a CDSS for predicting postoperative adverse outcomes may be challenged by inadequate generalizability across a variety of surgical populations. In this study, we test the hypothesis that models trained on one surgical cohort may be successfully applied to other cohorts that underwent different surgical procedures, by training them on the cardiac surgery cohort and testing them on vascular and acute non-cardiac surgery cohorts. The results obtained in this work suggest such generalizability and serve as a prototype for a CDSS where the ML models trained in one surgical cohort can be successfully applied to others.

Additionally, one should consider ensembles of different ML models in implementation of a CDSS, since no model definitively stands out as the best model in all cases. For example, in Cohort 3, RF has the highest performance of 0.82 in the 0.5- and 1-hour prediction windows, SVM of 0.81 in the 2-hour window, and LUCCK of 0.77 (which is equal to that of RF but with a smaller SD) in the 4-hour window.

As for limitations, sufficient data for training were only available for Cohort 1, allowing us only to train the models on Cohort 1 and test on Cohorts 2 and 3, but not vice versa. Adjusting the positive/negative case ratio to increase sensitivity may result in more false positives. Also, all cases in this study were from a single quaternary care center. The care processes and patient populations at other facilities can be substantially different. Another limitation is the few negative cases in Cohort 3, resulting in very high incidence rates of adverse events that are unlikely to be encountered in actual clinical settings. In future work, more non-cardiac surgical cases from other surgical care facilities would be needed to further increase the generalizability and robustness of these models. Also, our results must be interpreted with caution given that they were based on all surgical cohorts and may not necessarily extrapolate to non-surgical cohorts.

## Conclusion

In this study, we utilized DSP techniques such as TS estimation and DTCWPT to generate features from physiological waveforms. A novel tensor dimension reduction algorithm successfully reduced the feature space while still demonstrating translatable performance across different surgical cohorts. Four different types of ML models were trained on a cardiac surgery cohort and tested on different surgery cohorts. The RF models perform the best in terms of the AUROC, but LUCCK models maintain the most consistent range of performance, especially when the sample sizes are small. Our study suggests that ML models trained on a combination of waveform and EHR data of one group of surgical cases to predict life-threatening cardiovascular complications have potential to be successfully applied to other types of surgical cases, opening doors to the clinical decision support system enabling early detection of such events and timely interventions to improve clinical outcomes on a wide variety of surgical cases.

## Supplementary Information

Supplementary Information.

## Data Availability

The datasets generated or analyzed in this study were collected by Michigan Medicine. The University of Michigan’s Innovation Partnerships (UMIP) office will handle potential charges/arrangements of the use of data by external entities, using such methods as material transfer agreements. The executable code developed in this work is available from the corresponding author upon UMIP approval.
