# Supplementary material for: Prediction of postoperative cardiac events in multiple surgical cohorts using a multimodal and integrative decision support system
Source: Sci Rep. 2022 Jul 5;12:11347. doi: 10.1038/s41598-022-15496-w (PMC9256604; doi:10.1038/s41598-022-15496-w)
Supplement: Supplementary file 1 — Supplementary Information. [file 41598_2022_15496_MOESM1_ESM.docx]

Supplementary Tables for EHR Data

These tables provide more information about EHR Data features. In the following tables, the term “Retrospective” features are defined as follows: features from four additional retrospective periods of EHR data are collected before the decompensation event of a given individual. Each retrospective period is 4 hours long if the prediction window was less than 8 hours, and 8 hours if the prediction window was 8 hours or longer.

## Supplementary Table S1: Lab Values

Lab values are represented as the encoded Values “Low”, “Normal”, “High”, or “Critical”, defined in the table’s reference range.

| Lab Value | Reference Range | Unit | Includes Retrospective |
| --- | --- | --- | --- |
| Creatinine | Female: 0.5 – 1.0 Male: 0.7 – 1.3  Critical: > 2.0 | mg/dL | Yes |
| Glucose | 70 – 180 Critical: < 40 | mg/dL | Yes |
| Hematocrit | Female: 36 – 48 Male: 40 – 50 Critical: < 21 | % | Yes |
| Hemoglobin | Female: 12 – 16 Male: 13.5 – 17 Critical: < 7 | g/dL | Yes |
| International Normalized Ratio | 0.9 – 1.2 Critical: > 2.0 |  | Yes |
| Lactate | Arterial: 0.5 – 1.6 Venous: 0.5 – 2.2 Critical: > 4.0 | mmol/L | Yes |
| Platelet Count | 150 – 400 Critical: < 50 | 10^9^/L | Yes |
| Potassium | 3.5 – 5.0 Critical: > 6.0 | mmol/L | Yes |
| Sodium | 136 – 146 Critical: > 155 | mmol/L | Yes |
| White Blood Cell Count | 4 – 10 Critical: > 20 | 10^9^/L | Yes |

## Supplementary Table S2: Cardiovascular Infusions

Cardiovascular infusions (CVIs) are encoded as “Not on CVI”, “Normal CVI”, and “Elevated CVI”. Thresholds for “Normal” and “Elevated” are given in the Escalation Threshold column.

| Cardiovascular Infusion | Escalation Threshold | Includes Retrospective |
| --- | --- | --- |
| Dobutamine | 2.0 µg/kg/min | Yes |
| Dopamine | 2.5 µg/kg/min | Yes |
| Epinephrine | 0.02 µg/kg/min | Yes |
| Isoproterenol | 2.0 µg/kg/min | Yes |
| Milrinone | 0.25 µg/kg/min | Yes |
| Norepinephrine | 0.1 µg/kg/min | Yes |
| Vasopressin | 2.0 µg/kg/min | Yes |

## Supplementary Table S3: Vital Signs

Vital signs are extracted from EHR data for each tumbling window, or from the retrospective periods, and are reported as a numeric value.

| Vital Signs Feature | Includes Retrospective |
| --- | --- |
| Heart Rate | Yes |
| Mean Arterial Pressure | Yes |
| Respiratory Rate | Yes |
| SpO2 | Yes |
| Temperature | Yes |

## Supplementary Table S4: Additional EHR Data Features

These are additional EHR Data features that do not fall into the previous three categories.

| EHR Data Feature | Representation | Includes Retrospective |
| --- | --- | --- |
| Daily Intubation | Not Intubated / Intubated / Data Missing | No |
| Fraction of Inspired Oxygen | Numeric | No |
| Intubated | True / False | No |
| Positive End-Expiratory Pressure | Numeric, cmH_2_O | No |
| Urine Output | Numeric, mL | No |
